# Metabolic Impact of Body Fat Percentage Independent of Body Mass Index in Women with Obesity Remission After Gastric Bypass

**DOI:** 10.1007/s11695-019-04304-6

**Published:** 2019-12-12

**Authors:** Daniel Eriksson Hogling, Jesper Bäckdahl, Anders Thorell, Mikael Rydén, Daniel P. Andersson

**Affiliations:** 1 Department of Medicine (H7), Karolinska Institutet, Karolinska University Hospital, Huddinge, 141 86 Stockholm, Sweden; 2 Department of Clinical Sciences, Danderyd Hospital & Department of Surgery, Ersta Hospital, Karolinska Institutet, 116 91 Stockholm, Sweden

**Keywords:** Bariatric surgery, Body mass index, Metabolic syndrome, Insulin sensitivity, Body fat, X-ray absorptiometry

## Abstract

**Background/Objective:**

Body mass index (BMI) is central when evaluating treatment effect after gastric bypass. The metabolic impact of BMI-independent differences in body fat percentage (BF%) after gastric bypass is not fully understood. We compared metabolic and adipose tissue characteristics in women with high versus low BF% independent of BMI after obesity remission following gastric bypass.

**Subjects/Methods:**

A cohort of 215 women was included at baseline. A total of 166 women were re-examined 2 years after gastric bypass, whereof 130 had obesity remission (BMI < 30 kg/m^2^). Anthropometric parameters, blood pressure, and lipids were measured. Total and regional body fat mass was determined by dual-energy X-ray absorptiometry. Insulin sensitivity was assessed by homeostasis model assessment of insulin resistance (HOMA-IR) and hyperinsulinemic euglycemic clamp (*M* value). Adipocyte size and number were determined.

**Results:**

Of the 130 women with obesity remission, 64 had BF% ≥ 35 and 65 < 35. Independent of BMI, high BF% were associated with higher HOMA-IR (*P* = 0.021), lower *M* value (*P* = 0.0046), higher triglycerides (*P* = 0.013), higher visceral/total and android/gynoid fat mass ratios (*P* = 0.0032 and 0.0003 respectively), and larger subcutaneous fat cell volume (*P* < 0.0001) 2 years after gastric bypass. No differences in anthropometric measures, glucose, blood pressure, or fat cell number were observed.

**Conclusions:**

Independent of BMI, patients with higher BF% displayed lower insulin sensitivity, higher triglyceride levels, central fat distribution, and larger subcutaneous adipocytes 2 years after gastric bypass. Thus, determination of BF% provides additional information of metabolic characteristics at follow-up of non-obese patients after gastric bypass.

## Introduction

Body mass index (BMI) is the standard anthropometric measure for the categorization of overweight and obesity [1]. However, the use of BMI entails several limitations since body fat content is insufficiently captured by BMI. High body fat percentage (BF%), often defined as ≥ 35% in women, is reported in up to 50% of the population with a BMI within the non-obese range [2–5] and is linked to worse metabolic parameters [3, 6] including disturbed glucose metabolism [7] and future risk of type 2 diabetes [8].

Bariatric surgery, such as Roux-en-Y gastric bypass (RYGB), remains the most effective treatment of obesity [9–11]. Selection criteria for bariatric surgery have been virtually unchanged since the early 1990s and are still based on BMI and comorbidity [12, 13]. Postoperative BMI is central in the evaluation of treatment effect, and is the most frequently used measure to define outcome after bariatric surgery in randomized trials [14].

Weight loss induces favorable changes including improvement of metabolic parameters and adipose tissue function. Following RYGB, these improvements have been reported not only to be a normalization, but rather to reach “supranormal” levels [15, 16]. Weight loss–independent metabolic improvements are seen after RYGB, mediated by factors such as gut hormones and bile acids [17]. Whether these favorable changes attenuate the impact of excess BF% among patients in the non-obese range after bariatric surgery is not fully understood. In this study, we investigated if BMI-independent differences in BF% are associated with differences in metabolic function, anthropometric measures, or adipose tissue characteristics in patients reaching a non-obese state after RYGB.

## Materials and Methods

### Clinical Cohort and Surgical Procedure

The cohort studied herein was combined from two clinical trials (NCT01785134 and NCT01727245 at clinicaltrials.gov) with a similar study design, and main outcomes have been reported [18, 19]. Baseline characteristics of the entire cohort before and after weight loss have been presented previously [20]. Subjects with insulin, glitazone, or glucocorticoid treatment were excluded. All patients underwent RYGB. The length of the Roux-limb (alimentary limb) was typically 120 cm and the biliopancreatic limb 50 cm.

### Clinical Examinations and Determination of Fat Cell Size

All patients were investigated in the morning after fasting overnight. Anthropometric measures were obtained and BMI and waist-to-hip ratio (WHR) calculated. Subcutaneous adipose tissue samples were obtained by needle aspiration in the paraumbilical region under local anesthesia. Fat cell volume was determined through light microscopy after collagenase digestion as previously described [21]. Fat cell number in the android subcutaneous adipose tissue region (as defined below) was calculated by dividing the subcutaneous fat mass with average fat cell mass. Insulin sensitivity was determined in two ways: the homeostasis model assessment of insulin resistance (HOMA-IR) [22] and by hyperinsulinemic euglycemic clamp as described [23], quantified as mean glucose infusion rate during steady state plasma glucose and insulin concentrations (*M* value). Briefly, a 1.6 units/m^2^ body surface area bolus dose of insulin was administered intravenously, followed by a continuous intravenous infusion of insulin (Actrapid, Novo Nordisk, Copenhagen, Denmark) at a rate of 0.12 units/m^2^ body surface area/min during 2 h accompanied by a 200 mg/ml variable glucose infusion to maintain euglycemia (4.5–5.5 mmol/l). For normalization of the *M* value for lean body mass, lean body mass was calculated as body weight (kg) minus DXA-measured body fat mass (kg). Enzyme-linked immunosorbent assay was used to determine serum insulin levels (Mercodia, Uppsala, Sweden)**.** Plasma concentrations of glucose, cholesterol, triglycerides (TG), high-density lipoprotein (HDL) cholesterol, and apo B/apo A1 ratio were analyzed by the chemistry laboratory at the Karolinska University Hospital**.** The Friedewald’s formula was used to calculate low-density lipoprotein (LDL) cholesterol [24]**.** An automatic device (Omron M10-IT, Omron health care, Hoofddorp, the Netherlands) was used to measure blood pressure.

### Determination of Body Fat Mass and Distribution

Dual-energy X-ray absorptiometry (DXA, GE Lunar iDXA, GE Healthcare, Madison, WI, USA) was used to determine body fat mass, BF%, and body fat distribution. The enCore software (version 14.10.022, GE Healthcare, Madison, WI, USA) enabled determination of android and gynoid fat mass. The software defines the android region between the pelvis cut line, a line 20% of the distance between the pelvis and neck cut line and laterally by the arm cut line. Visceral fat mass can be estimated by the CoreScan feature (GE Medical Systems, Chalfont St. Giles, UK). The software assesses estimated visceral adipose tissue (EVAT) mass within the android region by subtracting the android subcutaneous fat from the total android fat. Thus, EVAT only refers to the visceral fat in the android region. Calibration checks of the DXA machine were performed daily.

### Statistical Analyses

Continuous variables are described as mean ± standard deviations. For between-group comparisons, unpaired two-sided *t* test was used for normally distributed parameters and Mann-Whitney *U* test for parameters not normally distributed. Normal distribution was assessed by Shapiro-Wilks test. Multiple regression analyses were used to adjust for residual differences in BMI and age, and to compare different independent variables versus *M* value. In the former analyses, an interaction term between the independent variables was tested for. *P* values < 0.05 were considered statistically significant. JMP Version 13 (SAS Institute Inc., Cary, NC, USA) was used for all statistical analyses.

## Results

### Clinical Characteristics

**Fig. 1:**
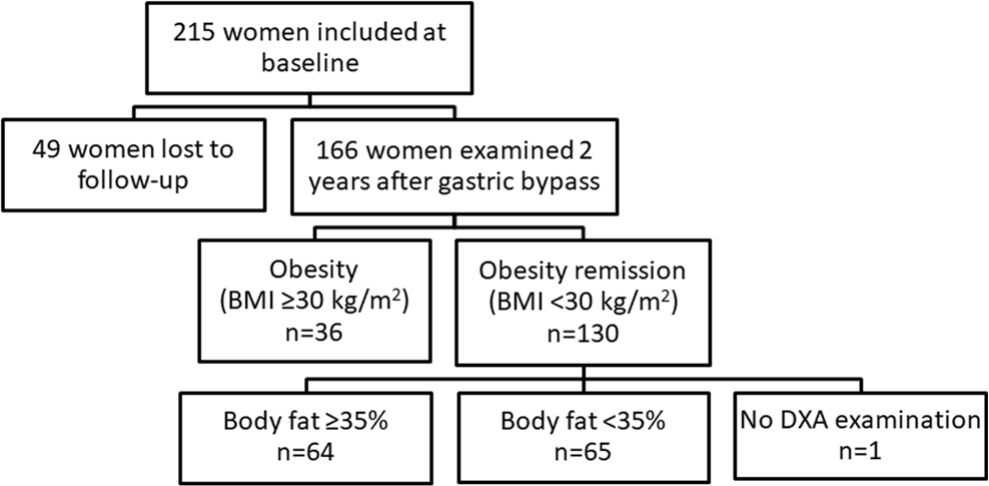
Flow chart of patients included in the study. BMI, body mass index; DXA, dual-energy X-ray absorptiometry

**Fig. 2:**
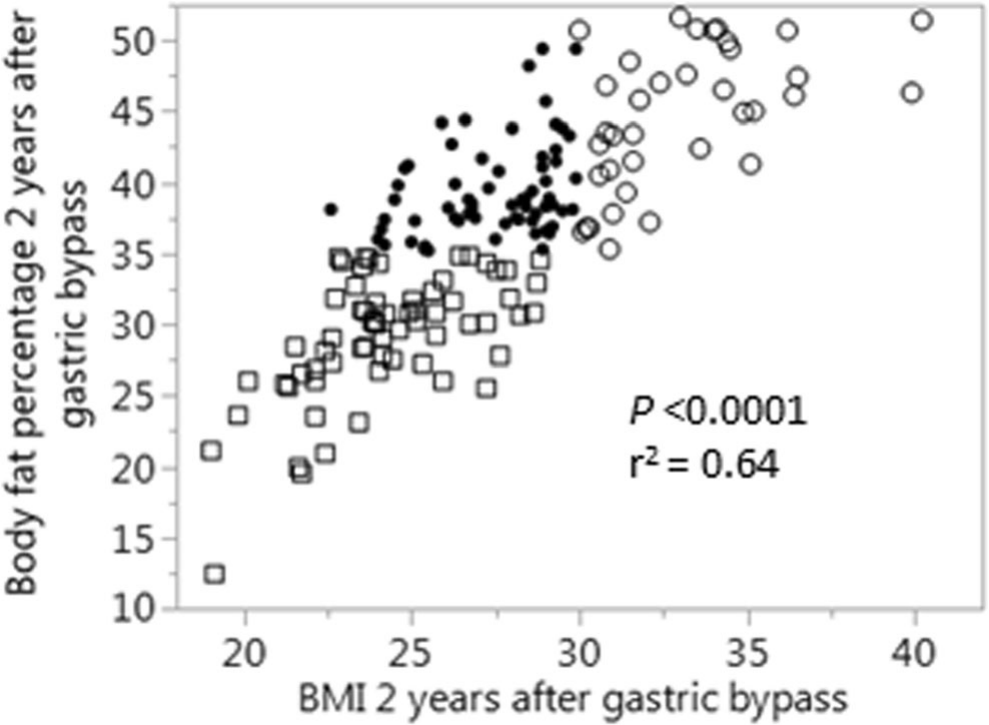
Body mass index (BMI) in relation to body fat percentage (BF%) 2 years after gastric bypass. Squares = BF% < 35; dots = BF% ≥ 35 and BMI < 30 kg/m^2^, circles = BMI ≥ 30 kg/m^2^. *P* and *r*^2^ values from linear regression analysis

A total of 215 women were included at baseline before surgery, and 166 (77.2%) were followed up 2 years after RYGB. Clinical characteristics of the cohort (*n* = 166) at baseline and after weight loss have been reported previously [20]. Mean BMI at baseline was 40.5 ± 4.2 kg/m^2^ and age 42.6 ± 9.5 years. A total of 130 women had achieved obesity remission (BMI < 30 kg/m^2^) at follow-up. Of the 130 women with obesity remission, 64 had BF% ≥ 35 and 65 had BF% < 35, while one woman did not perform the DXA examination (Fig. 1). The relationship between BMI and BF% after RYGB shown in Fig. 2 illustrates the BMI-independent variance in BF%. BF% at baseline for the 130 patients with obesity remission at follow-up was 51.6 ± 3.7 (52.5 ± 3.7 for the high BF% and 50.9 ± 3.5 for the low BF% group, *P* = 0.015). Before RYGB, 35 of the 130 patients with obesity remission at follow-up were on anti-hypertensive treatment, three patients treated with metformin, and eight with lipid-lowering drugs. At the follow-up, 18 had anti-hypertensive drugs, none remained on metformin, and three still had lipid-lowering treatment. A total of 20 patients had undergone omentectomy in addition to RYGB. Omentectomy in addition to RYGB has been shown not to affect metabolic outcome [18, 19]. In patients with obesity remission, data on *M* value was missing in 21 individuals, and data on fat cell volume in eight patients.

### Metabolic Profile and Adipose Tissue Characteristics in Women with High Versus Low Body Fat Percentage 2 Years After RYGB

**Fig. 3:**
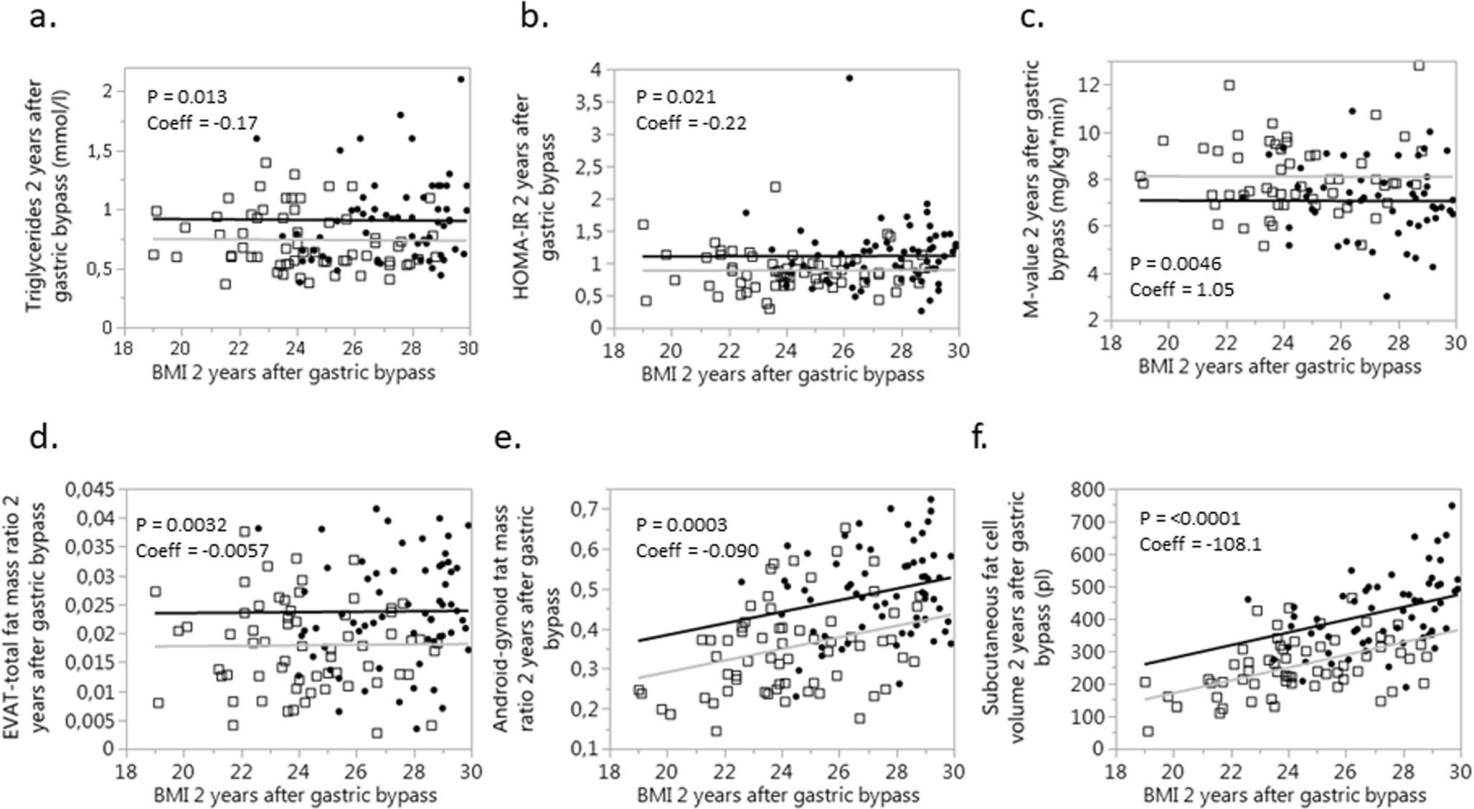
Triglyceride concentration (**a**), insulin sensitivity (**b**, **c**), body fat distribution (**d**, **e**), and subcutaneous fat cell volume (**f**) in women with high (≥ 35%, dots/black line) versus low (< 35%, squares/gray line) body fat percentage 2 years after gastric bypass, with body mass index as covariate in multiple regression analyses. *P* values and coefficients for body fat percentage. EVAT, estimated visceral adipose tissue; HOMA-IR, homeostasis model assessment of insulin resistance

None of the women with obesity (BMI ≥ 30 kg/m^2^) at follow-up 2 years after RYGB had BF% < 35 (Fig. 2), and only women with obesity remission were included in further analyses. Clinical, metabolic, anthropometric, and adipose parameters were compared between those with high BF% (≥ 35) and low BF% (< 35) and are presented in Table 1. Unadjusted analyses showed that the group with higher fat mass was characterized by older age, higher weight, BMI, glucose, insulin, HOMA-IR, total cholesterol, waist circumference, waist-to-hip ratio, android-to-gynoid and EVAT-to-total fat mass ratios, and larger subcutaneous fat cell volume and number as well as lower *M* value expressed per total body mass but not per lean body mass (Table 1). After adjustment for the difference in BMI using multiple regression analyses, patients with obesity remission and high BF% had higher insulin concentration (*P* = 0.044), HOMA-IR (*P* = 0.021), TG concentration (*P* = 0.013), subcutaneous fat cell volume (*P* < 0.0001), and android-to-gynoid as well as EVAT-to-total fat mass ratios (*P* = 0.0032 and 0.0003 respectively), and the *M* value expressed per body mass remained lower (*P* = 0.0046), see Table 1 and Fig. 3. No statistically significant interactions were observed between independent variables (except for age where *P* = 0.20 after including interaction), and interaction was therefore not considered in the adjusted model. Patients that were on medication for hyperlipidemia were excluded from the adjusted analyses of blood lipids, and patients with anti-hypertensive treatment were excluded from analyses of blood pressure. No statistically significant differences remained concerning age after adjustments for BMI, but after adjusting for both age and BMI in multiple regression analyses, the differences in HOMA-IR (*P* = 0.013), *M* value per total body mass (*P* = 0.049), subcutaneous fat cell volume (*P* < 0.0001), android-to-gynoid ratio (*P* = 0.0006), and EVAT-to-total fat mass ratio (*P* = 0.012) remained statistically significant, but not for insulin (*P* = 0.056). Given that no patient with low body fat had a BMI > 28.8 kg/m^2^, and only one patient with high body fat had BMI below 24 kg/m^2^ (see Figs. 2 and 3), we also performed analyses of BF% versus the statistically significant outcomes in the group with BMI 24–28.8 kg/m^2^ (*n* = 75). BMI did not differ significantly between those with low and high body fat percentage in this group (*P* = 0.19), but the between-group differences were still seen for triglycerides (*P* = 0.018), *M* value (*P* = 0.0044), EVAT-to-total fat mass ratio (*P* = 0.011), android-to-gynoid ratio (*P* = 0.0007), and fat cell volume (*P* < 0.0001) but not for HOMA-IR (*P* = 0.091). To investigate the impact on insulin sensitivity (*M* value), separate multiple regression analyses were performed with the statistically significant adipose tissue parameters (subcutaneous adipocyte volume and android-to-gynoid and EVAT-to-total fat mass ratios) combined with high/low BF% and BMI as covariates (Table 2). These analyses showed that android-to-gynoid fat mass ratio (*P* = 0.0008) and subcutaneous fat cell volume (*P* = 0.0013) predicted *M* value independently of BMI and BF%, whereas EVAT-to-total fat mass ratio did not (*P* = 0.20).

**Table 1:** Clinical and adipose tissue characteristics of non-obese patients with high versus low body fat percentage 2 years after RYGB

Clinical characteristics 2 years after RYGB	Body fat < 35% 2 years after RYGB (*n* = 65)	Body fat ≥ 35% 2 years after RYGB (*n* = 64)	Unadjusted^a^	Adjusted^b^	
			*P* value	*P* value	
				Body fat %	Interaction^c^
Age, years	43.0 ± 8.7	46.4 ± 10.4	0.044	0.10	0.024
Weight, kg	66.7 ± 7.7	74.5 ± 7.7	< 0.0001	0.94	0.92
BMI, kg/m^2^	24.2 ± 2.4	27.4 ± 2.4	< 0.0001	*–*	–
Omentectomy, *n*	9	11	0.63^d^	0.85	0.95
Glucose, mmol/l	4.9 ± 0.4	5.1 ± 0.5	0.030	0.17	0.077
Insulin, mU/l	4.1 ± 1.4	4.9 ± 2.1	0.0011	0.044	0.95
HOMA-IR	0.89 ± 0.31	1.11 ± 0.50	0.0017	0.021	0.63
*M* value, mg/kg × min	8.1 ± 1.5	7.1 ± 1.5	0.0006	0.0046	0.54
*M* value, mg/kg lean body mass × min	11.4 ± 2.3	11.4 ± 2.4	0.89	0.51	0.37
Triglycerides, mmol/l	0.76 ± 0.28	0.91 ± 0.34	0.0066	0.013	0.080
Cholesterol, mmol/l	3.9 ± 0.7	4.2 ± 0.9	0.021	0.088	0.11
HDL cholesterol, mmol/l	1.59 ± 0.31	1.67 ± 0.45	0.44	0.74	0.97
LDL cholesterol, mmol/l	1.96 ± 0.58	2.15 ± 0.63	0.059	0.17	0.12
Apo B/apo A1 ratio	0.53 ± 0.15	0.56 ± 0.19	0.41	0.14	0.055
Systolic blood pressure, mmHg	119.8 ± 15.1	121.3 ± 15.2	0.62	0.85	0.18
Diastolic blood pressure, mmHg	73.0 ± 11.7	74.6 ± 8.6	0.39	0.80	0.091
Waist circumference, cm	85.5 ± 6.6	93.8 ± 7.1	< 0.0001	0.18	0.16
Waist-to-hip ratio	0.88 ± 0.05	0.90 ± 0.06	0.013	0.87	0.14
Body fat, %	29.3 ± 4.4	39.5 ± 3.3	< 0.0001	< 0.0001	0.080
Total body fat, kg	19.1 ± 4.2	28.4 ± 3.9	< 0.0001	< 0.0001	0.98
Android-to-gynoid fat mass ratio	0.36 ± 0.12	0.49 ± 0.11	< 0.0001	0.0003	0.57
EVAT-to-total fat mass ratio	0.018 ± 0.0078	0.024 ± 0.0092	0.0002	0.0032	0.28
Subcutaneous fat cell volume, pl	255.7 ± 84.9	423.7 ± 113.9	< 0.0001	< 0.0001	0.15
Fat cell number within ESAT region, × 10^7^	403.7 ± 158.4	463.5 ± 150.8	0.027	0.75	0.33

Absolute values (mean ± standard deviations) and *P* values, unadjusted and adjusted for BMI. *BMI* body mass index, *ESAT* estimated subcutaneous adipose tissue, *EVAT* estimated visceral adipose tissue, *HOMA-IR* homeostasis model assessment of insulin resistance, *RYGB* Roux-en-Y gastric bypass

^a^Unpaired 2-sided *t* test used when normally distributed otherwise Mann-Whitney *U* test

^b^Multiple regression analyses adjusting for BMI (patients with anti-hypertensive treatment or lipid-lowering medication excluded when applicable)

^c^*P* value for interaction between body fat% and BMI

^d^2-tailed Fisher’s exact test

**Table 2:** BMI-independent impact of body fat percentage and adipose tissue covariates as determinants of insulin sensitivity. Multiple regression analyses with *M* value (mg/kg × min) as dependent variable versus body fat percentage (BF%, ≥ or < 35), body mass index (BMI), and different covariates as independent variables. All variables measured 2 years after gastric bypass in women with obesity remission. *P* values/standard beta coefficients (std beta). *EVAT* estimated visceral adipose tissue

Covariate	Covariate	BF%	BMI
	*P* value/std beta	*P* value/std beta	*P* value
Android-to-gynoid fat mass ratio, *n* = 108	0.0008/− 0.37	0.098	0.38
EVAT-to-total fat mass ratio, *n* = 108	0.20	0.019/0.28	0.99
Subcutaneous fat cell volume, *n* = 100	0.0013/− 0.43	0.19	0.14

## Conclusion

In this study, we demonstrate that high BF% in women with obesity remission after RYGB is associated with reduced insulin sensitivity, higher TG concentration, and a more unfavorable body fat distribution, independent of BMI. No statistically significant differences in fasting glucose, waist circumference, or waist-to-hip ratio were observed between patients with low and high BF%. These findings indicate that patients with high BF% may not be identified by these measures or BMI at follow-up after RYGB.

Several cross-sectional studies have reported on the discrepancies between BMI and BF% [4]. However, to the best of our knowledge, only one study has previously investigated this phenomenon in the long-term follow-up after RYGB [25]. In that study, Gómez-Ambrosi et al. compared BMI and BF% versus metabolic parameters after RYGB [3]. In line with our findings, they reported higher visceral fat content and TG concentration among women with higher body fat, but not with higher BMI. However, they also found differences in total and LDL cholesterol between women with low versus high body fat which were not observed in our study. Moreover, Gómez-Ambrosi et al. also included patients within the obese BMI range which may have influenced their results. Further differences between the studies were that our study focused on the BMI-independent impact of high BF% in the non-obese range after RYGB, that we used DXA to measure BF% and body fat distribution, and that we determined adipocyte size and number and measured insulin sensitivity by hyperinsulinemic clamp.

Although BMI is an easily assessed anthropometric measure, the findings herein display the limitations of this variable as a proxy for excess fat accumulation and its metabolic effects after bariatric surgery. The clinical impact of the between-group difference in insulin sensitivity observed at the follow-up 2 years after surgery may seem limited, with relatively small differences and average insulin sensitivity values still within the normal range in both groups. However, weight regain and deteriorated glucose control might be expected another couple of years after RYGB [9, 15]. In the long term, patients with high BF% may therefore be particularly vulnerable to recurrence of disturbed glucose metabolism or incident type 2 diabetes, a matter of further investigation in longitudinal cohorts. Moreover, patients with higher body fat displayed larger subcutaneous fat cells size which is known to predict the development of type 2 diabetes [26, 27].

We have previously reported that adipose tissues after weight reduction to a non-obese state display a more benign phenotype than in matched controls [16]. Many weight loss–independent mechanisms after RYGB, including incretin effects [17], are known to contribute to metabolic improvement and the metabolic impact of excess fat mass in the post-obese state after RYGB could therefore be limited. However, our findings show that relative fat mass remains a determinant of insulin sensitivity in the post-obese state, independent of BMI and age. Since no difference in insulin stimulated glucose uptake was found when normalized for lean body mass, differences in muscle mass are probably a contributor to the differences in whole body glucose disposal rate observed. However, a less favorable fat distribution and larger subcutaneous fat cell size may also be of importance. Our results demonstrate that larger subcutaneous fat mass depot is explained by adipocyte hypertrophy, i.e., increased fat cell size but not number, a phenomenon known to be linked to lower insulin sensitivity [23, 28]. In contrast to the observed differences in insulin sensitivity, body fat mass did not seem to be a major determinant of blood pressure or blood lipid levels in these post-obese patients. These disturbances may instead be more dependent on other factors.

The strengths of this study include that a relatively large and well characterized cohort was studied, with insulin sensitivity determined by two measures with consistent results. Furthermore, we used DXA to determine total and regional fat mass. The US Food and Drug Administration has approved this assessment of visceral fat for clinical use, and it is validated against reference methods such as computed tomography [29]. Limitations of this study include the fact that only women were investigated. Unfortunately, we have not been able to recruit enough men to allow for sex-specific analyses which would also require different cut-offs for high/low BF% in men. When interpreting the results, one should also bear in mind that the vast majority of women in this cohort were not diagnosed with type 2 diabetes before surgery. It is thus not known whether the findings could be generalized to men or patients with type 2 diabetes undergoing RYGB. The rationale behind using the 35% body fat cutoff point has been discussed [30] since there are no universally accepted definition of obesity based on body fat mass [4]. However, this threshold has been commonly used to define obesity based on body fat in previous studies [2, 3].

To conclude, higher BF% not reflected by BMI in the non-obese state after RYGB is accompanied by lower insulin sensitivity and higher triglyceride levels in women. Low muscle mass, android fat accumulation, and large subcutaneous fat cells may contribute to the decreased insulin sensitivity observed. Determination of insulin sensitivity, BF%, or body fat distribution at the follow-up after RYGB in women may add information on the metabolic status in women that have reached a non-obese state after RYGB. Whether patients with high body fat percentage independent of BMI after RYGB may be more prone to redevelopment of metabolic disturbances is a matter for future studies.
